# Microstructural Abnormalities in Children with Post-traumatic Stress Disorder: A Diffusion Tensor Imaging Study at 3.0T

**DOI:** 10.1038/srep08933

**Published:** 2015-03-11

**Authors:** Du Lei, Lingjiang Li, Lei Li, Xueling Suo, Xiaoqi Huang, Su Lui, Jing Li, Feng Bi, Graham J. Kemp, Qiyong Gong

**Affiliations:** 1 Huaxi MR Research Center (HMRRC), Department of Radiology, West China Hospital of Sichuan University, Chengdu, PR China; 2 Mental Health Institute, The Second Xiangya Hospital of Central South University, Changsha, PR China; 3 Departments of Psychiatry and Oncology, State Key Laboratory of Biotherapy, West China Hospital of Sichuan University, Chengdu, China; 4 Magnetic Resonance and Image Analysis Research Centre (MARIARC) and Institute of Ageing and Chronic Disease, University of Liverpool, United Kingdom; 5 Department of Psychology, School of Public Administration, Sichuan University, Chengdu, PR China

## Abstract

Posttraumatic stress disorder (PTSD) is a severe anxiety disorder characterized by re-experiencing, avoidance and hyperarousal. Brain microstructure abnormalities in PTSD, especially in children, are not yet well characterized. The aim of this study was to use MR diffusion tensor imaging (DTI) to identify brain microstructure alterations in children with PTSD compared to non-PTSD controls who experienced the same time-limited trauma. We studied 27 children with PTSD and 24 age- and gender-matched traumatized controls without PTSD, who all experienced the 2008 Sichuan major earthquake. DTI data were acquired and analyzed in terms of fractional anisotropy (FA), mean diffusivity (MD), radial diffusivity (RD) and axial diffusivity (AD). Children with PTSD showed an abnormal pattern, not only of FA, but also of the diffusivity measures MD, AD and RD. Most of the abnormal brain regions belonged to two important networks: the default-mode network, including precuneus and angular gyrus, and the salience network, including insula, putamen and thalamus. This DTI study identifies microstructural abnormalities of children with PTSD after a major earthquake, our results are consistent with the suggestion that pediatric PTSD is accompanied by a connectivity disequilibrium between the salience and default-mode networks, a finding of potential pathophysiological significance.

Posttraumatic stress disorder (PTSD) is a severe anxiety disorder developing after direct experience of a traumatic stressor, characterized by re-experiencing, avoidance and hyperarousal. It is not uncommon (lifetime prevalence is reported as 1–14% (American Psychiatric Association, 2000), and can have long-term consequences, notably in children, in whom psychological effects of trauma can persist into adulthood^1^.

Magnetic resonance imaging (MRI) has been used to quantify brain structural and functional abnormalities in PTSD. Diffusion tensor imaging (DTI) is an MRI technique used to characterize white matter microstructure by exploiting the diffusion characteristics of tissue water molecules^2^. DTI has been proved to be reliable and repeatable^3^,^4^, and has been widely used to investigate microstructural abnormalities in psychiatric disorders such as major depressive disorder (MDD)^5^,^6^,^7^, schizophrenia^8^,^9^,^10^ and ADHD^11^,^12^,^13^, as well as PTSD^14^,^15^,^16^. DTI data can be analyzed in several ways. Fractional anisotropy (FA), which quantifies the directionality of diffusion, is a fairly non-specific biomarker of microstructural architecture and neuropathology^17^. Mean diffusivity (MD) measures the average diffusion in all directions and provides information about changes in the interstitial space^18^. More neurobiological specificity is available from two directional diffusivities: although there are some important technical qualifications and caveats^19^, axial diffusivity (AD), which measures diffusion parallel to the axonal fibers, is correlated with axonal injury^20^ or axonal pruning^21^, while radial diffusivity (RD), which measures diffusion perpendicular to the fibers, is related to myelin injury or decreased myelination^22^,^23^.

We have recently published a meta-analysis of gray matter abnormalities in PTSD^24^. Results of the few studies of white matter abnormalities are diverse (for a recent review see Ref. 25), with reports of decreased FA in prefrontal cortex (PFC)^26^, anterior cingulum^26^,^27^,^28^ and posterior cingulum^29^, and increased FA in anterior gulum^30^ and superior frontal gyrus^28^. The early studies used manual tracing in predefined regions of interest^31^,^32^, which relies on anatomically specific prior hypotheses^33^. This bias is avoided by whole brain voxel-based analysis^34^, but in the few voxel-based studies of PTSD sample sizes are often small^26^,^27^,^28^,^29^,^30^,^35^, and results are potentially confounded by use of non-traumatized individuals (rather than similarly-traumatized non-PTSD individuals) as controls^36^, or by psychotropic medication^26^,^30^.

Children are thought to be more vulnerable to developing PTSD than adults^37^. To our knowledge, only one study has used DTI to investigate children with PTSD^15^. However, this is limited to maltreated children, which may have somewhat different psychological consequences than single-incident trauma^16^. Thus, the microstructural and microstructure networks abnormalities in the brains of children of PTSD have not been thoroughly investigated.

In this study, therefore, we used statistical parametric mapping (SPM) to perform a whole-brain analysis of the DTI data and to investigate patterns of whole-brain microstructural alterations in children who survived an 8.0-magnitude major earthquake, both those who had been diagnosed with PTSD, and those who had not (‘non-PTSD’) as controls. The potential power of the study to illuminate the neuropathophysiology of PTSD is enhanced by the unique characteristic of the trauma event, the relatively homogeneous demographic characteristics of the child trauma survivors, and the use of trauma-exposed non-PTSD controls. As well as comprehensive investigation of FA using the whole brain voxel-based analysis and rigorous statistical thresholds, we also studied the relationship of abnormalities in MD, RD and AD to clinical measures of symptom severity.

## Results

The detailed demographic and clinical characteristics of the participants are presented in Table 1. There were no significant between-group differences in the age, gender distribution or duration of school education.

**Figure 1:**
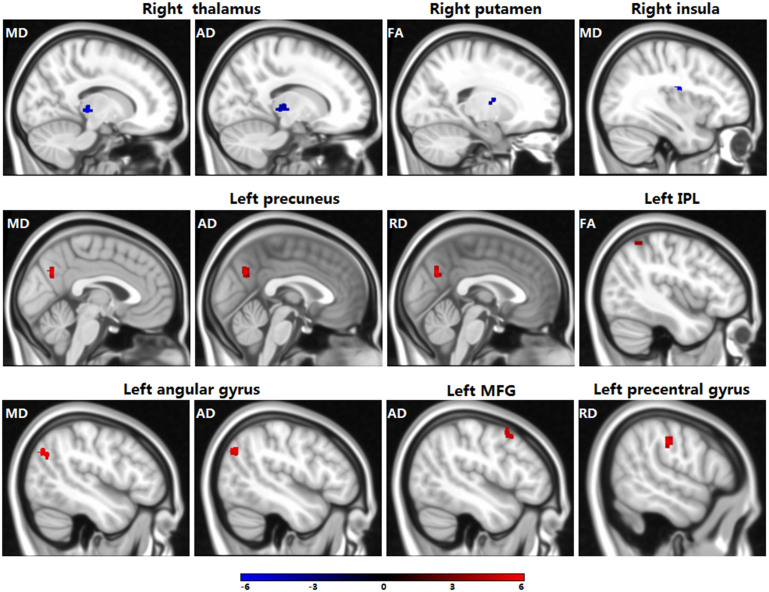
Compared to traumatized controls, children with PTSD showed significant changes in diffusion parameters. Abbreviations: IPL, inferior parietal lobule; MFG, middle frontal gyrus.

Compared to non-PTSD controls, children with PTSD demonstrated significant changes in the right putamen (decreased FA), left inferior parietal lobule (increased FA), right thalamus (decreased MD and AD), right insula (decreased MD), left precuneus (increased MD, AD and RD), left angular gyrus (increased MD and AD), left middle frontal gyrus (increased AD), and left precentral gyrus (increased RD). The detailed results are shown in Figure 1 and Table 2.

**Figure 2:**
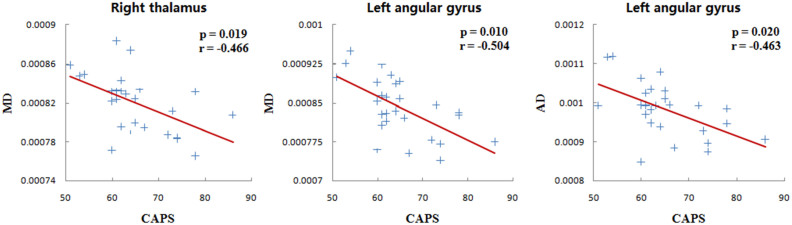
Brain regions showing relationships between DTI measures and clinician-administered PTSD scale (CAPS) scores in PTSD patients.

CAPS scores in PTSD patients were negatively correlated with mean MD in the thalamus (r = −0.446, p = 0.019) and angular gyrus (r = −0.504, p = 0.01), and with mean AD values in angular gyrus (r = −0.463, p = 0.02), after controlling for age and gender (Figure 2).

## Discussion

The present study explores white matter abnormalities in child survivors of a major earthquake, comparing those diagnosed with and without PTSD 10–15 months after this single but prolonged trauma, using not only the conventional DTI parameter FA, but also MD, AD and RD, which reflect different aspects of the underlying neurobiology.

In our study child patients with PTSD showed significantly decreased MD and AD in the right thalamus. The thalamus is a relay center between several subcortical areas and the cerebral cortex, subserving both sensory and motor mechanisms^38^. It is also implicated in memory, which can be influenced by stress^39^. There is neuroimaging evidence that thalamic dysfunction plays a part in dysregulation of emotional memories in PTSD. Functional MRI (fMRI) studies have shown that, compared with non-PTSD controls, adult PTSD patients showed less activation of the thalamus during internal generation of memories of the traumatic experience^40^. There is a case report of thalamic infarct resulting in the onset of adult PTSD with re-experience and reawakening of old traumatic memories^41^. Consistent with such a causal role, we found a negative correlation between CAPS scores and MD of right thalamus: that is, mean MD of right thalamus is decreased in child PTSD, and across individual patients, the higher the score (the worse the symptoms) the lower the MD (the worse the abnormality).

In the children with PTSD we also found decreased FA in right putamen, which is part of the basal ganglia. Structures of the basal ganglia play a role not only in the modification of movement, but also in cognition and emotion^42^. An fMRI study in normal adult subjects has revealed the role of the basal ganglia in enhancing working memory through attentional control and filtering of irrelevant information^43^. An fMRI study using a task requiring regulation of emotional reactions has found decreased activation in putamen in adult PTSD compared to healthy subjects^44^. Our results are consistent with this idea of a role for dysfunction of the putamen in the abnormal neural mechanisms in PTSD.

We also found decreased MD in right insula in children with PTSD. The insula is proposed to play a key role in the process by which increased prediction signal of a prospective aversive body state triggers an increase in anxious affect, worrisome thoughts and other avoidance behaviors^45^. Consistent with our finding, other neuroimaging work, including a recent meta-analysis of morphometry studies^46^ and a report of reduced gamma-aminobutyric acid (GABA) in the right anterior insula^47^, has suggested that the insula may be important in the abnormal neural mechanisms of adult PTSD^48^,^49^.

We found increased MD, AD and RD in the left precuneus in children with PTSD. Dysfunction of the precuneus, demonstrable using fMRI, is thought to be related to abnormalities of working memory in adult PTSD^50^. Interestingly, compared to controls, adult PTSD patients show greater activation of the precuneus in response to trauma-related stimuli^51^.

We also found increased MD in the angular gyrus in children with PTSD, which was negatively correlated with CAPS scores (r = −0.504, p = 0.01). MD is the sum of AD (axial diffusion) and RD (radial diffusion), and further analysis revealed significant changes only of AD (p = 0.02), suggesting that it is changes of AD in the angular gyrus which contribute to this negative correlation with disease severity. This finding is of interest as it suggests the possibility that increased AD may mitigate severity of disease in this population, as reported in schizophrenia^52^, Gulf War illness^53^ and Alzheimer's disease^54^. The angular gyrus is believed to play a role in organizing language and thoughts^55^ and increased neural activity in the left angular gyrus has been observed in patients with PTSD^56^. Increased AD reflects faster water movement along the axons, which might be functionally associated with enhanced neural transmission. We therefore hypothesize that the MD increase may be compensatory.

The precuneus and angular gyrus are commonly considered as the central node of the default-mode network (DMN)^57^. Parts of the DMN have exhibited functional abnormalities in PTSD in previous neuroimaging studies^58^,^59^,^60^. It might be that the increased MD and AD we observed in these DMN regions (which is, for precuneus at least, negatively correlated with symptom score) is related to a strengthened role in coordinating whole-brain networks as part of compensatory response to the pathological disorder of PTSD.

The other areas in which we found microstructural abnormalities, namely the insula, putamen and thalamus, are all components of the salience network (SN), which plays an important role in attention, cognition, control, working memory and switching^61^,^62^,^63^. In contrast to the DMN regions (precuneus and angular gyrus), in these salience-network areas we observed a decrease in FA, MD or AD. It has been proposed that functional alterations of PTSD involve a triple network model which corresponds broadly with the salience network, central executive network, and default model network^64^,^65^. Taken together, our findings are compatible with an earlier study^60^ which proposed that disequilibrium between the salience network and the DMN is important in the pathophysiology of PTSD.

The present sample is homogeneous for surviving a single traumatic event, and all of the children are drug-naïve, which provides a good opportunity to observe disease-related changes in brain and behavior without confounds. Several issues remain to be addressed. First, our results show some difference in gray matter, such as thalamus. It is generally accepted that FA is more sensitive in white matter because of the specialized structure of nerve fibers. However, FA, MD, AD, and RD can also be used to measure microstructure connections of dendrites and soma in gray matter, and DTI has been used to detect microstructure changes of gray matter^66^,^67^,^68^,^69^. Thus our results suggest that dysfunction of gray matter, as well as of white matter, may also play a role in PTSD pathophysiology. Second, limited by the relatively small number of gradient orientations in our DTI acquisition, we did not attempt fiber tracking. Further studies with more gradient directions in MRI acquisition are encouraged to explore the neurocircuitry in PTSD using direct connectivity analyses. Third, this study is cross-sectional; whether the microstructural abnormalities evolve dynamically remains to be established in longitudinal studies. Fourth, studies of functional connectivity using fMRI will be needed to confirm the suggestion of a connectivity disequilibrium between the salience and default-mode networks in PTSD. Finally, in order to control for the traumatic stress-related brain alterations^70^, we chose the population who were also exposed to the major earthquake but did not develop PTSD as controls. Future studies using non-traumatized healthy participants as controls will provide further insights into PTSD pathophysiology.

## Conclusion

This DTI study identifies the microstructural abnormalities of children PTSD patients exposed to a major earthquake. Our findings demonstrate regional microstructural abnormalities with an altered pattern of diffusion changes, not only FA, but also specific directional diffusivities (MD, AD and RD), in a group of highly homogeneous single-incident traumatized individuals with pediatric PTSD. Most of the brain regions with alerted DTI measures in children with PTSD were the components of two important networks: the default-mode network (DMN), including precuneus and angular gyrus, and the salience network (SN), including insula, putamen, and thalamus. Our results are consistent with the suggestion that pediatric PTSD is associated with a disequilibrium between the salience network and the DMN. This may give new insights into structural network abnormalities in PTSD, assisting our understanding the pathogenesis of this disorder.

## Methods

### Subjects

We recruited 51 child (≤16 years) survivors of the 2008 Sichuan earthquake 10–15 months after the event. To be included, survivors must have (i) physically experienced the earthquake, and (ii) personally witnessed death or serious injury or the collapse of buildings, but (iii) suffered no physical injury or serious head trauma with any known cognitive consequences, or any loss of consciousness >5 min. Exclusion criteria, assessed using Structured Clinical Interview for DSM-IV (SCID), included: any history of or current psychiatric or neurological disorders other than PTSD, with the exception (to avoid an unrepresentative sample) of current depression, panic disorder, and generalized anxiety deemed secondary to PTSD; drug abuse; current psychotropic medication use (to avoid possible confounding effects on brain structure); previous psychiatric diagnosis; and IQ < 80. Each participant was interviewed and screened with the PTSD checklist (PCL)^71^. Survivors scoring ≥35 were given the Clinician-Administered PTSD Scale(CAPS)^72^ by psychiatrists to confirm the PTSD diagnosis. Finally 27 children with PTSD and 24 non-PTSD individuals were included for DTI assessment. The detailed demographics and clinical characteristics of the participants are presented in Table 1. The study protocol was designed in accordance with guidelines outlined in the Declaration of Helsinki and approved by the Research Ethics Committee of the Huaxi Hospital of Sichuan University. After a complete description of this study was provided to the parents of each subject, the informed consent was obtained and every child agreed to participate.

### Data acquisition

MRI data were acquired on a 3T MR imaging system (EXCITE; General Electric) using a single-shot spin-echo echo planar image (SE-EPI) sequence. The diffusion sensitizing gradients were applied along 15 non-collinear directions (b-value = 1000 s/mm^2^) together with an acquisition without diffusion weighting (b = 0). Imaging parameters were: TR = 12000 ms, TE = 71.6 ms, number of excitations (NEX) = 2, slice thickness = 3 mm, 50 slices, 128 × 128 matrix and 24 × 24 cm field of view (FOV).

### Data processing and statistical analysis

SPM 8 (http://www.fil.ion.ucl.ac.uk/spm/), MATLAB 2010 (The Math Works, Natick, MA) and FSL4.1 (http://www.fmrib.ox.ac.uk/fsl/) were used to analyze the data. First, the DICOM files of each DWI acquisition were converted into a single multivolume NIFTI file. FSL's eddy current correction was then applied. The brain was extracted using BET (Brain Extraction Tool, http://fsl.fmrib.ox.ac.uk/fsl/bet2/). Finally, FA, MD, RD and AD maps were calculated using FSL.

The correction of head motion image artifacts, normalization and statistical analyses were performed using SPM 8. We developed a customized pediatric template using SPM to reduce potential errors caused by matching to an adult template^73^. This development had three steps. First, we normalized the FA maps of the control group based on the deformation information generated from the corresponding unweighted images (first b = 0 image) and the echo-planar imaging (EPI) template (in MNI152 space). These, termed wFA maps, were averaged to produce a mean map which was smoothed (using a [6,6,6] FWHM Gaussian kernel) to obtain the customized pediatric template^74^. We normalized all of the original FA maps (from both the patient and control groups) to the deformation field produced from the original FA maps and our FA-specific pediatric template. The normalized FA maps were smoothed using a [6,6,6] filter for statistical analysis. Finally, a fractional design specification was set up to compare the PTSD group against the controls using a two-sample t-test. The same processes were applied to the MD, AD and RD maps. For all analyses, the statistical map cluster level threshold was set to p < 0.05 and voxels ≥ 45 (Alphasim corrected), determined through Monte Carlo simulations^75^ using AFNI AlphaSim (http://afni.nimh.nih.gov/afni/).

Significant clusters' mean DTI parameter values (FA, MD, AD and RD) were calculated using Marsbar-0.42.Then partial correlations were computed to examine relationships between the mean DTI parameter values in regions and CAPS score, using age and gender as covariates.

## Author Contributions

D.L., Li. L. and Q.G. designed the study and drafted the manuscript. Le. L., X.S., X.H., S.L., J.L. and F.B. performed the experiments. G.K. modified the manuscript. D.L. and Q.G. carried out statistical analyses. All authors reviewed the manuscript.

## Figures and Tables

**Table 1:** Demographics and Clinical Characteristics of Participants^a^

	NC (n = 24)	PTSD (n = 27)	p-value
Age^b^	13.2(±1.3)	13.0(±1.8)	0.76^d^
Gender (male/female)	11/13	11/16	0.71^e^
Handedness (R/L)	24/0	27/0	—
Years of education^c^	7.8(±1.6)	7.7(±1.7)	0.78^d^
Time since trauma (months)^b^	12.1(±1.6)	11.7(±1.6)	0.41^d^
PTSD checklist	24.0(±2.9)	53.8(±5.1)	—
CAPS	—	65.1(±8.3)	—

^a^Data are presented as mean ± SD. Analyses performed in SPSS 16.0 (http://www.spss.com). All tests were two-tailed. No significant differences were found between PTSD patients and non-PTSD controls in age, gender, education and time since trauma. Abbreviations: NC: traumatized controls; PTSD: patients with post-traumatic stress disorder; CAPS: clinician-administered PTSD scale.

^b^At the time of magnetic resonance scanning.

^c^Total years of completed education as reported by participant and guardian.

^d^By two-sample t-test.

^e^By Pearson chi-square test.

**Table 2:** Diffusion parameter results from voxel-based analysis of the PTSD group compared with the non-PTSD control group

Regions	t value^a^	Number of voxels	Hemisphere	Peak location^b^ (X Y Z)			Correlation^c^ (CAPS score)
***FA decrease***							
Putamen	3.81	47	Right	20	−4	12	−0.007(0.974)
***FA increase***							
Inferior Parietal Lobule/BA40**d**	2.7	47	Left	−42	−50	56	0.161(0.443)
***MD decrease***							
Thalamus	4.07	115	Right	14	−26	−2	**−0.446(0.019)**
Insula/BA 13	3.23	54	Right	36	−12	22	−0.001(0.996)
***MD increase***							
Precuneus/BA 7	5.13	65	Left	−2	−66	34	−0.086(0.683)
Angular Gyrus	3.47	55	Left	−46	−70	34	**−0.504(0.010)**
***AD decrease***							
Thalamus	4.6	176	Right	10	−24	0	−0.324(0.114)
***AD increase***							
Precuneus	5.48	70	Left	0	−64	32	−0.247(0.234)
Angular Gyrus	3.84	60	Left	−46	−72	34	**−0.463(0.020)**
Middle Frontal Gyrus/BA 6	3.72	61	Left	−48	12	50	−0.203(.330)
***RD increase***							
Precuneus	5.01	65	Left	0	−64	30	−0.047(.825)
Precentral Gyrus	3.99	49	Left	56	−20	38	−0.229(.272)

^a^All effects survived after alphasim correction(p < 0.05).

^b^For peak areas of activation; X, Y, Z = MNI coordinates.

^c^Data are presented as the correlation coefficient r (p-value), p < 0.05 are shown in bold font.

^d^BA: Brodmann area.
